# Apolipoprotein E gene polymorphism and renal function are associated with apolipoprotein E concentration in patients with chronic kidney disease

**DOI:** 10.1186/s12944-019-1003-x

**Published:** 2019-03-09

**Authors:** Monika Czaplińska, Agnieszka Ćwiklińska, Monika Sakowicz-Burkiewicz, Ewa Wieczorek, Agnieszka Kuchta, Robert Kowalski, Barbara Kortas-Stempak, Alicja Dębska-Ślizień, Maciej Jankowski, Ewa Król

**Affiliations:** 1 Clinic & Chair of Nephrology, Transplantology and Internal Medicine, Medical University of Gdańsk, Dębinki 7, 80-211 Gdańsk, Poland; 2 Department of Clinical Chemistry, Medical University of Gdańsk, Dębinki 7, 80-211 Gdańsk, Poland; 3 Department of Molecular Medicine, Medical University of Gdańsk, Dębinki 7, 80-211 Gdańsk, Poland; 4 Department of Therapy Monitoring and Pharmacogenetics, Medical University of Gdańsk, Dębinki 7, 80-211 Gdańsk, Poland

**Keywords:** Chronic kidney disease, Apolipoprotein E, Apolipoprotein E gene polymorphism, Lipoprotein

## Abstract

**Background:**

Chronic kidney disease (CKD) associates with complex lipoprotein disturbances resulting in high cardiovascular risk. Apolipoprotein E (APOE) is a polymorphic protein with three common isoforms (E2; E3; E4) that plays a crucial role in lipoprotein metabolism, including hepatic clearance of chylomicrons and very low-density lipoprotein (VLDL) remnants, and reverse cholesterol transport. It demonstrates anti-atherogenic properties but data concerning the link between polymorphism and level of APOE in CKD patients are inconclusive.

The aim of our research was to assess the relationship between *APOE* gene polymorphism and APOE concentration and its redistribution among lipoproteins along with CKD progression.

**Methods:**

90 non-dialysed CKD patients were included into the study. Real time PCR was used for *APOE* genotyping. APOE level was measured in serum and in isolated lipoprotein fractions (VLDL; IDL + HDL; HDL). Kidney function was assessed using eGFR CKD-EPI formula.

**Results:**

The population was divided into three *APOE* genotype subgroups: E2(ε2ε3), E3(ε3ε3) and E4(ε3ε4). The highest APOE level was observed for the E2 subgroup (*p* < 0.001). APOE concentration positively correlated with eGFR value in the E2 subgroup (r = 0.7, *p* < 0.001) but inversely in the E3 subgroup (r = − 0.29, *p* = 0.02).). A lower concentration of APOE in the E2 subgroup was associated with its diminished contents in HDL and IDL + LDL particles. In the E3 subgroup, the higher concentration of APOE was related to the increased number of non-HDL lipoproteins.

**Conclusion:**

In patients with CKD, *APOE* genotype as well as renal function are associated with the concentration of APOE and its redistribution among lipoprotein classes.

## Background

Chronic kidney disease (CKD) is one of the current leading public health problems due to increasing frequency, complications as well as high mortality resulting from accelerated atherosclerosis [1]. The main cause of death in this population is cardiovascular disease (CVD) connected with dyslipidaemia, which is observed at early stages of renal failure and associated with the degree of glomerular filtration rate declining [2, 3]. Hypertriglyceridemia, accumulation of intact or partially metabolised apolipoprotein B (APOB)-containing lipoproteins (very low-density lipoprotein (VLDL), intermediate density lipoprotein (IDL), low density lipoprotein (LDL)), and reduced concentration of high density lipoprotein (HDL) are well documented lipid disturbances in CKD [3–7]. Other studies have also shown that the concentration and distribution of apolipoproteins - which play a crucial role in lipid metabolism - are also disturbed in CKD [8].

Apolipoprotein E (APOE), a 34 kDa glycoprotein, is produced mainly in the liver but its local production has been documented in the brain, kidneys, spleen, adrenals and macrophages [9]. It is a component of all classes of lipoproteins except small dense LDL. It circulates between HDL and APOB-containing lipoproteins in plasma [10, 11]. APOE acts as a ligand for receptor-mediated clearance of chylomicrons (CM) and VLDL remnants from the circulation to the liver and takes part in reverse cholesterol transport (RCT) as a component of HDL [11]. The protective role of APOE in atherosclerosis development was first proven in animal models [12]. In further research it was demonstrated that APOE is an anti-atherogenic protein also in humans [9, 13, 14]. It was also shown that cholesterol-loaded macrophages demonstrated higher *APOE* gene expression; APOE deficiency in these cells decreased cholesterol efflux and led to atherosclerosis plaques formation [15].

There are three common APOE isoforms (APOE2, APOE3, APOE4) coded by three alleles (ɛ2, ɛ3, ɛ4) of the *APOE* gene on chromosome 19q13.2, therefore there are six *APOE* gene polymorphisms: *APOE2/2, APOE3/3, APOE4/4, APOE2/3, APOE3/4* and *APOE2/4* [11, 16]. The isoforms differ in two amino acids at residues 112 and 158 - APOE2 has cysteine at both sides, APOE4 has arginines at both sides and APOE3 has cysteine at 112 residue and arginine at 158 residue [11]. Due to the differences in APOE isoforms structure, they possess diverse binding capacity to receptors as well as lipoprotein-binding preferences that results in a various influence on the lipoproteins metabolism [11]. APOE3 and APOE4 isoforms bind to the LDL receptor with similar potent affinity, whereas APOE2 is defective in interaction with the receptor - only 2% of normal activity [16]. APOE2 and APOE3 isoforms preferentially bind to small HDL, while APOE4 to large triglyceride (TG)-rich VLDL particles [17]. APOE3 is the most common isoform (around 77% of the Caucasian population) and does not impair lipoprotein metabolism. APOE4 occurs in 15% of the population and can lead to elevated LDL-C concentration. APOE2 is the rarest isoform (8%) and might be related with hypertriglyceridemia [17, 18]. These disturbances in various ways contribute to accelerated atherosclerosis. The *APOE* gene polymorphism also influences APOE concentration in serum; the highest levels were measured in ε2 allele carriers, the lowest in ε4 carriers [18].

Although APOE has anti-atherogenic features, there are ambiguous data about the link between APOE concentration and CVD risk [19–21]. Surprisingly, it has been shown in many studies that CVD patients had elevated APOE concentration [18, 19, 22–24]. However, researchers have highlighted that APOE distribution between lipoproteins has a crucial meaning and total APOE concentration in serum is not an appropriate parameter for the evaluation of CVD risk. It has been proven that increased APOE level in the CVD population resulted from higher APOE content in APOB-containing lipoproteins and that factor could be a proper marker for this purpose [22, 25].

The important role of APOE in lipid metabolism and CVD development in CKD patients has also been emphasised by many researchers [22, 26, 27]. The polymorphism of the *APOE* gene has an influence on APOE concentration as well as lipids and lipoproteins disturbances. Wang Y et al. proved that hemodialysed ε4 allele carriers have higher total cholesterol (TC), TG, and LDL-C [27]. ε2 allele carriers develop accumulation of cholesterol-rich VLDL [28]. However, there is poor knowledge about the relationship between *APOE* gene polymorphism, APOE level, its distribution among lipoproteins and the degree of kidney dysfunction, especially at early stages of CKD. These findings could shed new light on the pathomechanism of lipid-related disorders and accelerated atherosclerosis in CKD patients.

The aim of our research was to assess the relationship between *APOE* gene polymorphism and APOE serum concentration and its redistribution among lipoprotein classes along with CKD progression.

## Materials and methods

### Study group

Ninety non-dialyzed adult CKD patients at the stage from 3a to 4 (eGFR 59-16 ml/min/1. 73m^2^) being under the care of the Outpatient Nephrology Clinic of the University Clinic Centre in Gdańsk (Poland) were included into the study. The exclusion criteria included: diabetes, liver diseases, infections, nephrotic proteinuria, active neoplasm, immunosuppressive treatment including steroids, heparin treatment due to lipoprotein lipase activation and hypolipidemic agents except statins.

The Independent Bioethics Commission for Research of the Medical University of Gdańsk (Poland) approved the study, performed in accordance with the Declaration of Helsinki and all the participants provided written informed consent.

Demographic data about medications, traditional risk factors of CVD (age, sex, smoking cigarettes, hypertension, obesity) and personal medical histories were collected during a medical appointment. During the physical examination 3-times blood pressure (BP) in a few minute intervals were measured with an Omron Upper Arm Blood Pressure Monitor. The first measurement was ignored and the mean value was calculated from the second and third measurements. Hypertension was defined as a systolic BP ≥ 140 mmHg and/or diastolic BP ≥ 90 mmHg in two medical appointments in the Outpatient Clinic and/or current antihypertensive treatment. Moreover, body mass index (BMI) and waist-to-hip ratio (WHR) were assessed. Obesity was diagnosed on the basis of a BMI ≥ 30 kg/m^2^.

### Methodology

Blood samples were obtained from the vein after an overnight fasting of a minimum of ten hours. VLDL was isolated from blood serum by ultracentrifugation [7, 29]. HDL was isolated by precipitation of APOB-containing lipoproteins with heparin and manganese chloride from the ultracentrifugal d > 1.006 g/ml fraction [30]. In the serum and in isolated VLDL and HDL fractions the concentration of lipids and apolipoproteins were measured. (IDL + LDL)-lipid and apolipoprotein levels were calculated as a difference: total concentration of component in serum - VLDL - HDL.

Serum total protein (TP), albumin, TC, TG and plasma glucose were measured using commercially available kits obtained from Pointe Scientific (Warsaw, Poland). Apolipoprotein AI, E and B were measured by immunonephelometry using kits obtained from Siemens Healthcare Diagnostics (Erlangen, Germany) [31, 32]. LDL-C was calculated using Friedewald formula.

Serum creatinine was measured using the enzymatic method that has calibration traceable to an IDMS reference measurement procedure (Abbott Diagnostics Inc., Santa Clara, CA, USA) [33]. CKD-EPI equation was used to estimate GFR.

Genomic DNA was isolated by manual extraction using columns from a commercially available kit obtained from A&A Biotechnology (Gdynia, Poland).

*APOE* genotyping (rs429358, rs7412) was performed by real time PCR reaction using MutaREAL® APOE real time PCR Kit obtained from Immundiagnostik AG (Bensheim, Germany) [34]. Briefly, for analysis of *APOE* genotyping (rs429358 or rs7412) the potentially mutated region of the *APOE* gene was amplified by PCR in a capillary by LightCycler (Roche, Basel, Switzerland) using genomic DNA. Amplification products were analyzed afterwards in a melting-point curve analysis with mutation specific hybridization probes detected at 670 nm. The melting-point curve analysis allowed a clear identification of wildtype, heterozygous or homozygous genotypes. For rs429358, rs7412 *APOE* genotyping the Hybridization Probes were designed in this way that their sequence fits exactly onto the CGC-sequence. Therefore, in the melting point curve analysis the mutation peak arose earlier in the presence of TGC-sequence because of the introduced mismatch (= sequences are not 100% homologous). In the case of heterozygous genotype two peaks were generated – one with a lower temperature (TGC) and one with a higher temperature (CGC).

### Statistical analysis

Continuous variables were expressed as a mean ± standard deviation (SD) or as a median and 10th – 90th percentiles, and were compared using the unpaired Student’s t-test, Mann-Whitney U test, or one-way analysis of variance, as appropriate. Categorical variables, expressed as a number (percent), were compared using χ^2^ test. Correlations were estimated using Spearman’s rank method. Statistical analyses were performed using STATISTICA PL 12.0 (Statsoft, Cracow, Poland). *P* value of < 0.05 was considered statistically significant.

## Results

### Characteristics of the study group

The baseline characteristics of the CKD patients are presented in Table 1. The study population was categorised into 3 APOE subgroups according to *APOE* gene polymorphism: subgroup E2 (ε2ε3 allele carriers), E3 (ε3ε3), and E4 (ε3ε4). In our research group there were neither homozygotes *APOE2–2* and *APOE4–4* nor *APOE2–4* heterozygotes. The analyzed subgroups did not differ in their age, sex, BMI index, status of smoking cigarettes, statin treatment, as well as kidney function measured by eGFR CKD-EPI value (Table 1). They also did not differ in their TP, albumin and glucose levels (Table 1).

**Table 1:** Baseline characteristic of the study groups

Parameter	Total	APOE subgroup			*P* value
		E2	E3	E4	
cases	90 (100%)	18 (20%)	58 (64%)	14 (16%)	NA
sex [F/M]	37/53	8/10	23/35	6/8	0.92**
age [years]	68 ± 10	65 ± 12	68 ± 11	66 ± 10	0.50*
hypertension	73 (81%)	17 (94%)	45 (78%)	11 (79%)	0.19**
obesity	27 (30%)	7 (39%)	15 (26%)	5 (36%)	0.50**
BMI [kg/m^2^]	28 ± 5	29 ± 7	27 ± 4	29 ± 5	0.27*
elevated WHR (≥ 1 for males, ≥ 0.85 for females)	45 (50%)	11 (61%)	26 (45%)	8 (57%)	0.58**
smoking cigarettes	40 (44%)	11 (61%)	23 (40%)	6 (42%)	0.28**
statin therapy	48 (53%)	11 (61%)	27 (47%)	10 (71%)	0.18**
G3a stage CKD	28 (31%)	4 (22%)	20 (35%)	4 (29%)	0.59**
G3b stage CKD	42 (47%)	7 (39%)	29 (50%)	6 (42%)	0.68**
G4 stage CKD	20 (22%)	7 (39%)	9 (15%)	4 (29%)	0.11**
creatinine [mg/dl]	1.6 (1.2–2.6)	1.7 (1.0–2.8)	1.6 (1.1–2.2)	1.6 (1.3–2.9)	0.24***
eGFR CKD-EPI [ml/min/1. 73m^2^]	39 ± 12	37 ± 13	41 ± 12	36 ± 13	0.34*
total protein [g/l]	64.1 ± 4.1	64.5 ± 4.3	63.7 ± 4.0	64.3 ± 4.5	0.49*
albumin [g/l]	42.9 ± 3.1	42.6 ± 3.3	43.0 ± 3.1	42.9 ± 2.7	0.90*
glucose [mg/dl]	95.0 (82.8–109.8)	95.3 (85.7–107)	94.4 (81.7–108.7)	96.7 (83–121)	0.86***

Continuous values are presented as mean ± standard deviation (SD) or median and range (10th and 90th percentiles). Differences among the subgroups were analysed using ANOVA*, Pearson’s chi-squared test**, or Kruskall-Wallis test***

BMI - body mass index, WHR - waist-to-hip ratio, CKD - chronic kidney disease, eGFR CKD-EPI - estimated glomerular filtration rate-chronic kidney disease-epidemiology collaboration

Subgroups: E2: ɛ2ɛ3 subjects; E3: ɛ3ɛ3 subjects; E4: ɛ3ɛ4 subjects

There were no significant differences in a basic lipid profile parameters as well as APOB and APOAI levels between the APOE subgroups (Table 2).

**Table 2:** Lipid parameters in the study groups

Parameter	Total	APOE subgroup			*P* value
		E2	E3	E4	
TC [mg/dl]	204 ± 48	201 ± 26	207 ± 54	198 ± 42	0.77*
Non-HDL-C [mg/dl]	154 ± 47	148 ± 26	156 ± 53	154 ± 43	0.65*
LDL-C [mg/dl]	130 ± 44	121 ± 22	134 ± 50	125 ± 39	0.29*
HDL-C [mg/dl]	50 ± 13	54 ± 15	50 ± 12	43 ± 10	0.31*
TG [mg/dl]	112 (64–182)	122 (64–241)	98 (63–164)	121 (69–277)	0.21**

Values are presented as mean ± SD or median and range (10th and 90th percentiles). Differences among the subgroups were analysed using ANOVA* or Kruskall-Wallis test**

TC - total cholesterol, LDL-C – low density lipoprotein-cholesterol, HDL-C – high density lipoprotein-cholesterol, TG - triglycerides, APOB - apolipoprotein B, APOAI - apolipoprotein AI, non-HDL-C - non-high density lipoprotein-cholesterol (very low-density-cholesterol + intermediate density lipoprotein-cholesterol + LDL-C)

Subgroups: E2: ɛ2ɛ3 subjects; E3: ɛ3ɛ3 subjects; E4: ɛ3ɛ4 subjects

### Concentration of APOE in serum and in lipoprotein fractions according to *APOE* genotype

There were statistically significant differences in total APOE and APOE-HDL concentrations as well as in APOE-HDL/APOAI ratio depending on *APOE* genotype (Table 3). In the E2 subgroup, the total APOE and APOE-HDL levels were significantly higher in comparison to the E3 (for total APOE: *p* < 0.05; APOE-HDL: *p* < 0.001; APOE-HDL/APOAI: *p* < 0.001) and E4 (for total APOE: *p* = 0.002; APOE-HDL: *p* < 0.001; APOE-HDL/APOAI: *p* < 0.001) subgroups (Table 3). There were no differences in the APOE-non-HDL, APOE-IDL + LDL and APOE-VLDL concentrations nor in the APOE/APOB ratios between the subgroups (Table 3).

**Table 3:** APOE, APOAI, and APOB concentrations [mg/dl] in serum and in lipoprotein fractions in the study groups

Parameter	Total	APOE subgroup			*P* value
		E2	E3	E4	
APOE	4.08 ± 1.27	4.94 ± 1.47	4.00 ± 1.15	3.34 ± 0.80	< 0.001*
APOE-HDL	1.45 ± 0.60	2.08 ± 1.15	1.38 ± 0.50	0.90 ± 0.34	< 0.001**
APOE-non-HDL	2.66 ± 0.93	2.86 ± 1.15	2.64 ± 0.92	2.44 ± 0.66	0.43
APOE-IDL + LDL	2.06 ± 0.85	2.23 ± 1.25	2.09 ± 0.72	1.70 ± 0.62	0.23
APOE-VLDL	0.57 ± 0.42	0.63 ± 0.52	0.51 ± 0.37	0.70 ± 0.44	0.23
APOAI	168 ± 30	178 ± 33	166 ± 30	164 ± 25	0.49
APOE-HDL/APOAI	0.0086 ± 0.0031	0.0117 ± 0.0019	0.0082 ± 0.0027	0.0056 ± 0.0023	< 0.0001***
APOB	94 ± 25	91 ± 11	95 ± 27	97 ± 30	0.95
APOB-IDL + LDL	85 ± 24	81 ± 12	85 ± 25	87 ± 37	0.97
APOB-VLDL	8.8 ± 4.2	9.4 ± 4.2	8.1 ± 4.0	10.7 ± 4.4	0.08
APOE/APOB in non-HDL	0.029 ± 0.0085	0.032 ± 0.012	0.029 ± 0.0074	0.026 ± 0.0068	0.40
APOE/APOB in IDL + LDL	0.025 ± 0.010	0.028 ± 0.015	0.025 ± 0.0082	0.021 ± 0.0073	0.15
APOE/APOB in VLDL	0.071 ± 0.044	0.070 ± 0.046	0.071 ± 0.044	0.073 ± 0.045	0.95

Values are presented as mean ± SD. Differences among the subgroups were analysed using ANOVA and test post hoc Tukey’s test

APOE - apolipoprotein E, APOAI - apolipoprotein AI, APOB - apolipoprotein B, non-HDL - non-high density lipoprotein (VLDL + IDL + LDL), HDL - high density lipoprotein, VLDL - very low-density lipoprotein, IDL - intermediate density lipoprotein, LDL - low density lipoprotein

Subgroups: E2: ɛ2ɛ3 subjects; E3: ɛ3ɛ3 subjects; E4: ɛ3ɛ4 subjects

* E2 vs E4 *p* = 0.002, E2 vs E3 *p* = 0.052, E3 vs E4 *p* = 0.291, ** E2 vs E4 *p* < 0.001, E2 vs E3 p < 0.001, E3 vs E4 *p* = 0.039, *** E2 vs E4 p < 0.001, E2 vs E3 p < 0.001, E3 vs E4 *p* = 0.016

### Relationship between eGFR CKD-EPI and APOE concentration in serum and in lipoprotein fractions according to *APOE* genotype

**Fig. 1:**
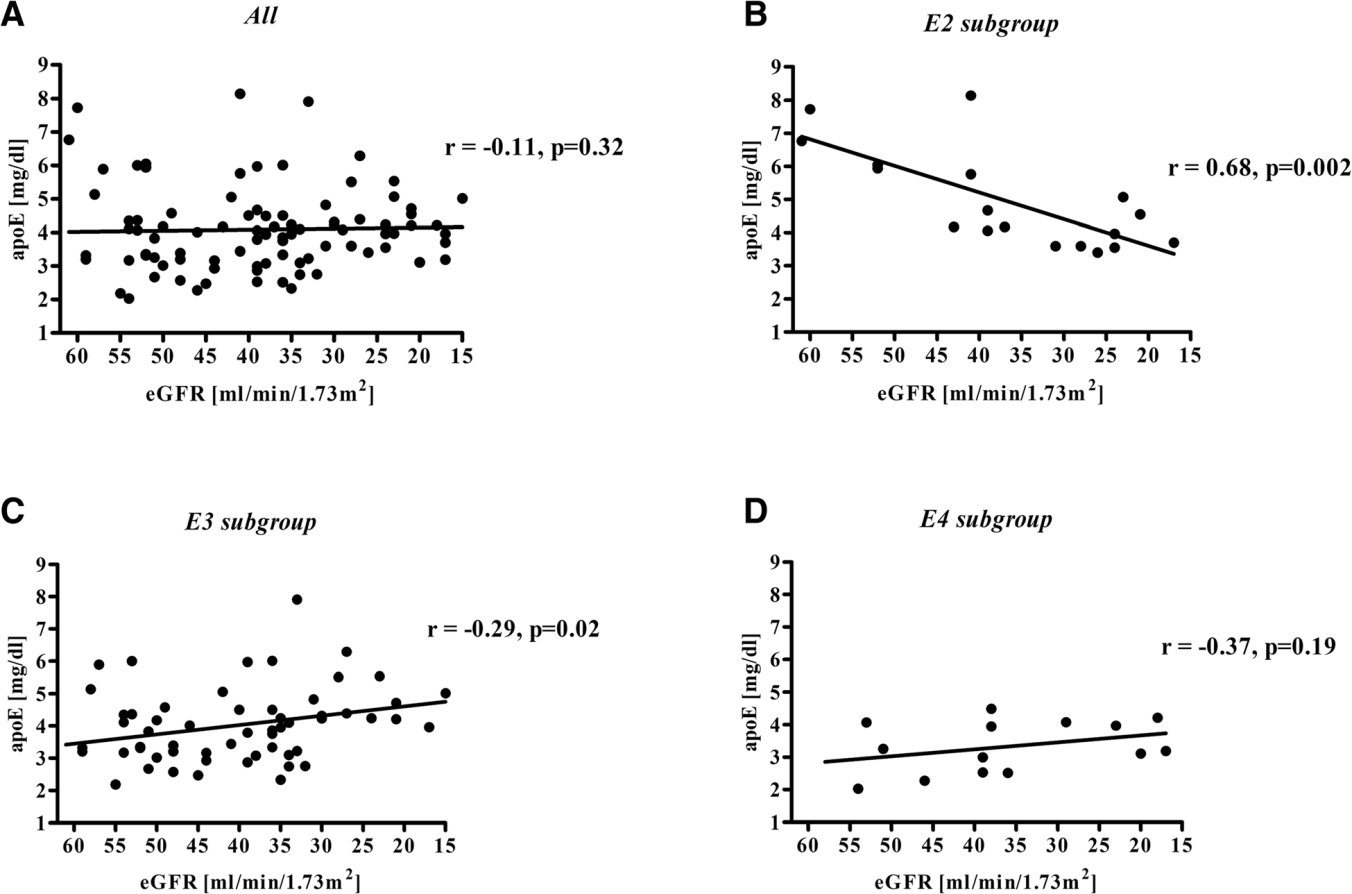
Correlation between eGFR and serum APOE concentration for the total study group (**a**) and subgroups: E2 (**b**), E3 (**c**), and E4 (**d**). Subgroups: E2: ɛ2ɛ3 subjects; E3: ɛ3ɛ3 subjects; E4: ɛ3ɛ4 subjects. APOE - apolipoprotein E, eGFR - estimated glomerular filtration rate

In the entire study group there was no correlation between APOE serum concentration and eGFR value (Fig. 1a, Table 4).

**Table 4:** Univariate correlation between eGFR CKD-EPI and APOE, APOAI, and APOB concentrations, and apolipoprotein ratios

Parameter	Total		APOE subgroup					
			E2		E3		E4	
	R	p	R	p	R	p	R	p
apoE	−0.11	0.32	0.68	0.002	−0.29	0.02	−0.37	0.19
apoE-HDL	−0.08	0.48	0.66	0.002	−0.15	0.27	−0.31	0.28
apoE-non-HDL	−0.08	0.47	0.57	0.01	−0.29	0.03	−0.25	0.38
apoE-IDL + LDL	0.09	0.39	0.78	< 0.001	−0.18	0.19	−0.18	0.54
apoE-VLDL	−0.26	0.02	−0.46	0.05	−0.19	0.15	−0.16	0.58
apoAI	0.10	0.34	0.64	0.004	−0.11	0.42	0.28	0.33
apoE-HDL/apoAI	−0.06	0.61	0.53	0.03	−0.11	0.43	−0.41	0.14
apoB	−0.20	0.06	0.05	0.83	−0.25	0.06	−0.38	0.18
apoB-IDL + LDL	−0.21	0.05	0.09	0.72	−0.28	0.04	−0.44	0.12
apoB-VLDL	−0.03	0.81	−0.10	0.69	0.07	0.61	0.13	0.67
apoE/apoB in non-HDL	0.15	0.18	0.56	0.01	−0.10	0.46	0.01	0.97
apoE/apoB in IDL + LDL	0.24	0.03	0.71	< 0.001	0.02	0.89	0.07	0.82
apoE/apoB in VLDL	−0.26	0.02	−0.34	0.17	−0.26	0.07	−0.17	0.56

APOE - apolipoprotein E, APOAI - apolipoprotein AI, APOB - apolipoprotein B, HDL - high density lipoprotein, VLDL - very low-density lipoprotein, IDL - intermediate density lipoprotein, LDL - low density lipoprotein

Subgroups: E2: ɛ2ɛ3 subjects; E3: ɛ3ɛ3 subjects; E4: ɛ3ɛ4 subjects

For the E2 subgroup, APOE serum concentration was positively correlated with eGFR value (Fig. 1b, Table 4); this relationship remained statistically significant in multiple linear regression after adjusting for sex, BMI, and statin therapy (β = 0.806; standard error (SE) = 0.184; *p* < 0.001). For the E3 subgroup, the APOE level was inversely correlated with eGFR value (Fig. 1c, Table 4) and this relationship also remained significant after adjustment for the above-mentioned factors (β = − 0.286; SE = 0.122; *p* = 0.02).

**Fig. 2:**
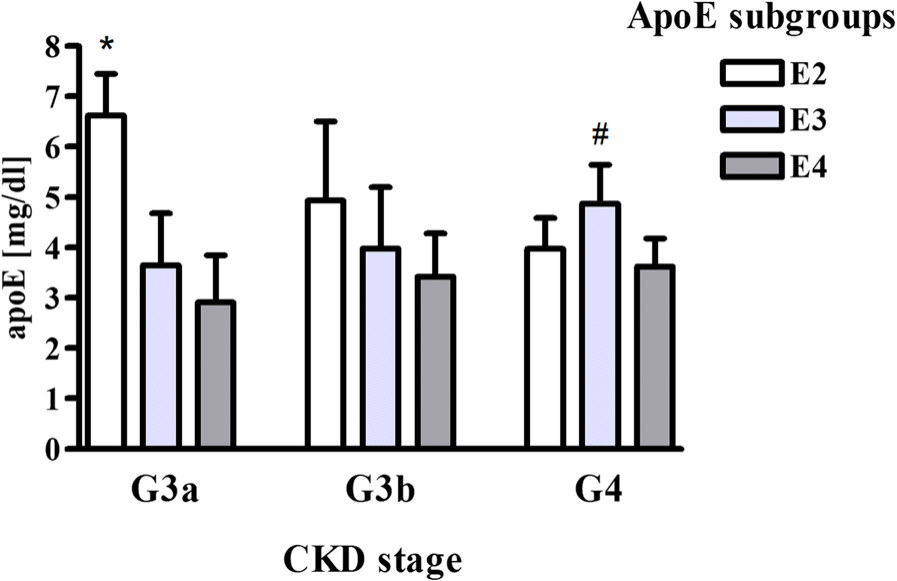
APOE serum level in CKD patients depending on *APOE* genotype and CKD stage. Subgroups: E2: ɛ2ɛ3 subjects; E3: ɛ3ɛ3 subjects; E4: ɛ3ɛ4 subjects. Data are expressed as mean ± SD. *p < 0.001 vs. E3 and E4 subgroups (G3a stage), #*p* < 0.05 vs. E2 and E4 subgroups (G4 stage). APOE - apolipoprotein E, CKD - chronic kidney disease

For the E2 subgroup there was lower APOE-HDL and APOAI concentrations, and APOE-HDL/APOAI ratio, along with a decline in eGFR (Fig. 2, Table 4). The same relationship was observed for APOE level and APOE/APOB ratio in IDL + LDL (Fig. 2, Table 4). For VLDL there was an opposite tendency; APOE-VLDL level negatively correlated with eGFR value(Table 4).

For the E3 subgroup, there was no relationship between APOE-HDL level and eGFR. Conversely, in non-HDL lipoproteins, the APOE and APOB concentrations increased along with eGFR decline, and the APOE/APOB ratio remained constant (Table 4).

For the E4 subgroup, we did not observe a statistically significant relationship between total APOE level in serum or individual lipoprotein fractions, and eGFR (Table 4).

**Fig. 3:**
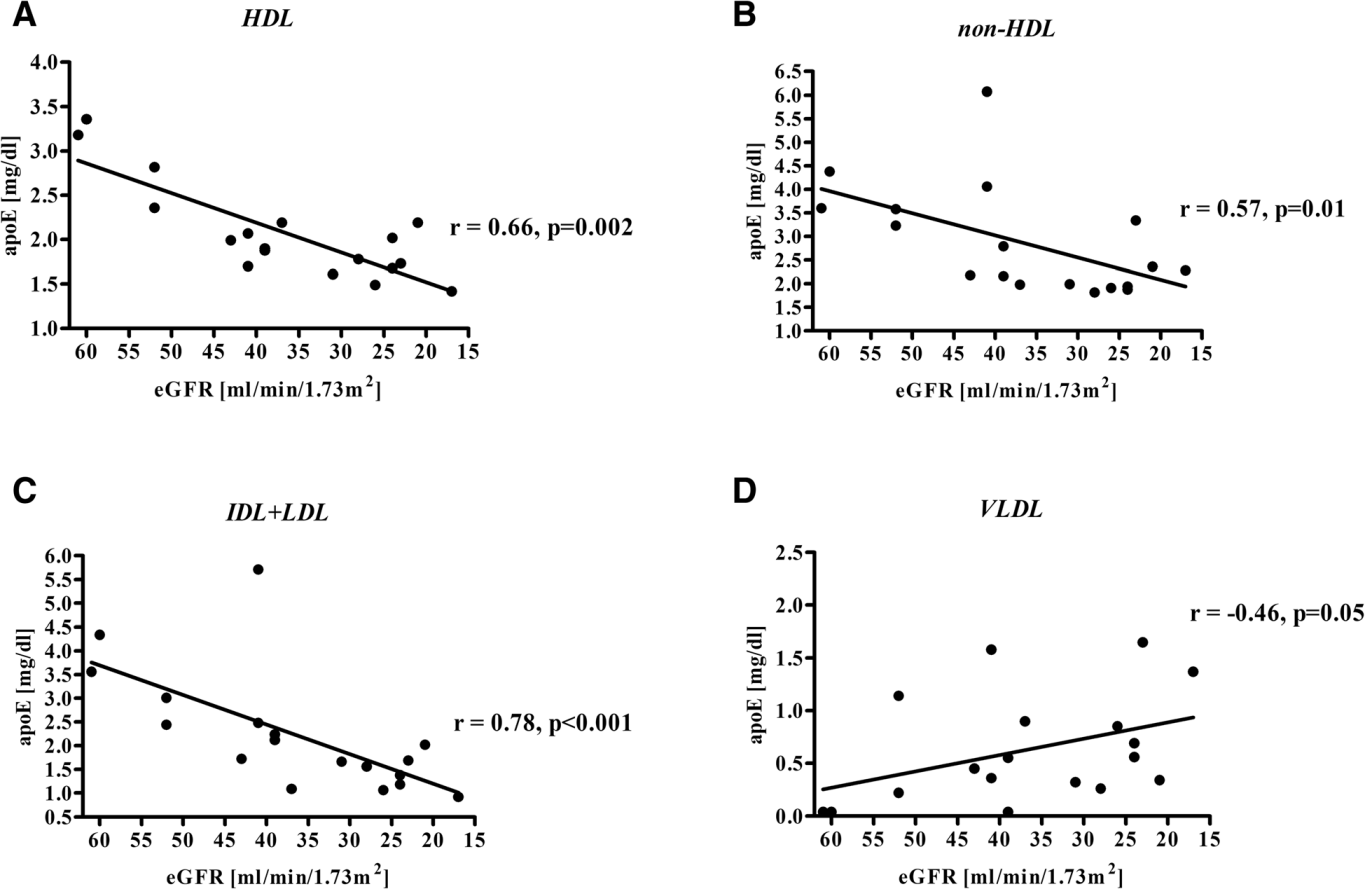
Correlation between eGFR and APOE concentration in HDL (**a**), non-HDL (**b**), IDL + LDL (**c**), and VLDL (**d**) for the E2 subgroup (ɛ2ɛ3 subjects). APOE - apolipoprotein E, eGFR - estimated glomerular filtration rate, HDL - high density lipoprotein, non-HDL - non-high density lipoprotein, IDL - intermediate density lipoprotein, LDL - low density lipoprotein, VLDL - very low density lipoprotein

After division of the APOE subgroups according to CKD stage, we observed statistically significant differences in APOE concentration (Fig. 3). In the G3a CKD stage APOE level was higher in the E2 subgroup (*p* < 0.001), whereas in the G4 CKD stage it was increased in the E3 subgroup (*p* < 0.05) compared to other subgroups (Fig. 3).

## Discussion

Our findings point to the association of *APOE* genotype with APOE concentration and its distribution between lipoproteins in CKD patients depending on kidney function.

Differences in APOE level according to APOE isoform were proven in previous studies, in both non-CKD and CKD patients [9, 22, 23, 27]. In accordance with other researchers we observed that APOE level was higher for the E2 subgroup in comparison with the E3 and E4 subgroups [27].

In our own and previous research, it was also observed that APOE concentration did not change with eGFR value decline in CKD patients [6, 35]. However, after adjusting the data for APOE isoform we have established that there is a correlation between APOE level and eGFR; in the ε2 allele carriers there was a decrease of APOE concentration with the decline of eGFR, while for the E3 subgroup there was a significant increase of APOE level. In the ε4 allele carriers, there was tendency of an increase in APOE level with eGFR drop, but the statistical significance was not reached. Thus we proved that there is an association between *APOE* gene polymorphism and APOE concentration in patients with CKD, during progression of renal dysfunction. To the best of our knowledge this is the first study presenting such a relationship.

For the E2 subgroup, higher APOE concentration in comparison to the E3 and E4 subgroups was observed in the G3a CKD stage. The high APOE level in the ε2 allele carriers is considered to be a beneficial mechanism, compensating for the decreased affinity of APOE2 to hepatic receptors [36]. Nonetheless, it should be noted that for the ε2 allele carriers in the G4 CKD stage, the APOE level was significantly lower in comparison with the G3a CKD stage patients, and it was similar or even lower in comparison to the E3 and E4 subgroups (Fig. 3). The mechanism of such a phenomenon should become a subject of further research.

To explain the reason of the correlations observed between APOE level and eGFR we analyzed the APOE level and calculated the APOE/APOAI and APOE/APOB ratios for individual lipoprotein fractions.

For the E2 subgroup, we observed a correlation between APOE levels and eGFR for all lipoprotein fractions. For IDL + LDL particles, APOE lowered with a decrease of eGFR. However, APOB - the quantitative marker of IDL and LDL particles in serum, did not decrease, thus we might conclude that the ε2 carriers in the G4 CKD stage had lower APOE level in IDL + LDL particles, in comparison to the G3a CKD stage patients, but the amount of these lipoproteins was constant along with the CKD progression. [37] (Table 4). The lower content of APOE in IDL + LDL can lead to suppression of the hepatic uptake of these lipoproteins since APOE is a ligand for hepatic LDLR and LRP receptors [38]. Thus, these disturbances could be related to prolonged IDL and LDL clearance from the circulation and their prolonged retention in the arteries walls, which predisposes to atherosclerotic plaques formation [10].

Conversely, the APOE level in VLDL particles increased with progression of renal dysfunction, which may lead to their delayed metabolism and, in a consequence, to hypertriglyceridemia since APOE inhibits lipoprotein lipase activity [39].

In HDL, both the qualitative and quantitative differences in the composition of particles were noticeable in the E2 subgroup for patients with various stages of renal dysfunction. The observed decrease of APOE and APOAI levels in these particles, together with a drop of APOE-HDL/APOAI ratio with the decline of eGFR, may suggest that the number of HDL particles in these patients diminished with the renal dysfuntion, especially those containing APOE. Such HDL disturbances could be related to accelerated atherosclerosis development and higher risk of incident CVD, since it has been proven that the lower amount of HDL as well as lower APOE content in HDL impair all steps of RCT [1, 38–40]. This is due to the fact that APOE participates in cholesterol uptake from the cells in the interaction with the ATP-binding cassette transporter (ABCG1). Moreover, it is an activator of acyltransferase lecithin:cholesterol (LCAT), which esterifies cholesterol in HDL and contributes in cholesteryl esters transmission from HDL to the hepatocytes via Scavenger receptors class B type 1 (SR-B1) and LDL receptors [13, 41]. In addition, HDL containing APOE are a reservoir of APOE for TG-rich lipoproteins. In the circulation, APOE is transferred from HDL to the IDL and LDL particles, allowing for their hepatic clearance [38]. Thus, it can be concluded that a decrease in APOE serum level and its redistribution among lipoprotein classes in ε2 carriers observed with eGFR decline can promote the progression of lipid disturbances and accelerate atherosclerosis development.

Contrary to the E2 subgroup, for the E3 subgroup APOE-non-HDL level (especially IDL + LDL) increased along with deterioration of renal function. We could suppose that ε3 homozygotes with more advanced kidney failure accumulated IDL + LDL particles in the plasma since an increase in APOB-(IDL + LDL) concentration with a decline of eGFR has been observed and the ratio of APOE/APOB in IDL + LDL remained constant. Such a phenomenon can be considered as one of the factors accelerating atherosclerosis development, since it has been shown in both non-CKD and CKD patients that APOE level in APOB-containing lipoproteins positively associates with incident CVD risk [22, 42]. Corsetti J et al. showed that high APOE level positively correlated with incident CVD but it referred only to APOE in APOB-containing lipoproteins [22]. Barbagallo CM et al. proved in a group of males with premature CVD that accumulation of TG-rich lipoproteins with elevated APOE content may comprise an additive factor potentially promoting and initiating the atherosclerotic process [25]. Van Vliet P et al. showed that in old aged patients high plasma APOE level was associated with a higher risk of stroke [21]. Thus, it can be concluded that for the patients from the E3 subgroup, the increase in APOE level and its contents in lipoproteins with CKD progression are also unfavourable, despite their being different than for the ε2 carriers.

We did not observe a statistically significant relationship between APOE level and CKD progression for the patients from the E4 subgroup, nevertheless there was a tendency of increase of APOE level with eGFR decline, similar to the E3 subgroup. The lack of statistically significant relationship could be related to the small number of participants in this subgroup, resulting from the low prevalence of the ε4 allele in the study group. The relatively small number of participants in our research was also due to the many exclusion criteria. However, we could eliminate the possible influence of other chronic illnesses, e.g. diabetes or treatment on APOE level and other lipid and apolipoprotein parameters. We also excluded metabolic syndrome and malnutrition in all of the examined subgroups, which could potentially influence lipid and lipoprotein metabolism. Nevertheless, it should be emphasised that the frequency of *APOE* gene polymorphism in our study group was comparable to APOE isoform occurrence in other studies in CKD and general populations [6, 14, 43].

## Conclusions

In summary, our results support earlier reports about the relationship between *APOE* gene polymorphism and APOE concentration. However, we have shown for the first time that in CKD patients, the APOE level and its distribution between lipoprotein classes is associated not only with *APOE* gene polymorphism but also with kidney function. Therefore, it can be concluded that lipid and lipoprotein disorders in CKD should be analyzed considering *APOE* gene polymorphism. Further observation is crucial to explain the role of the association between *APOE* gene polymorphism and lipid disturbances in CVD development in CKD, as well as to establish if there is a link between lipid disturbances for particular ε allele carriers and increased rate of CKD progression.

## Abbreviations

APO: apolipoprotein
BMI: body mass index
BP: blood pressure
CKD: chronic kidney disease
CKD-EPI: chronic kidney disease epidemiology collaboration
CM: chylomicrons
CVD: cardiovascular disease
eGFR: estimated glomerular filtration rate
HDL: high density lipoprotein
HTG: hypertriglyceridemia
IDL: intermediate density lipoprotein
LDL: low density lipoprotein
LDLR: low density lipoprotein receptor
LPR: LDL receptor-related protein
Non-HDL: non-high density lipoprotein (IDL + LDL)
PCR: polymerase chain reaction
RCT: reverse cholesterol transport
TC: total cholesterol
TG: triglyceride
TP: total protein
VLDL: very low-density lipoprotein
WHR: waist-to-hip ratio
